# Comparing end-effector position and joint angle feedback for online robotic limb tracking

**DOI:** 10.1371/journal.pone.0286566

**Published:** 2023-06-08

**Authors:** Mattia Pinardi, Alessia Noccaro, Luigi Raiano, Domenico Formica, Giovanni Di Pino

**Affiliations:** 1 NEXT: Neurophysiology and Neuroengineering of Human-Technology Interaction Research Unit, Università Campus Bio-Medico di Roma, Rome, Italy; 2 Neurorobotics Group, Newcastle University, Newcastle, United Kingdom; University rehabilitation institute, SLOVENIA

## Abstract

Somatosensation greatly increases the ability to control our natural body. This suggests that supplementing vision with haptic sensory feedback would also be helpful when a user aims at controlling a robotic arm proficiently. However, whether the position of the robot and its continuous update should be coded in a extrinsic or intrinsic reference frame is not known. Here we compared two different supplementary feedback contents concerning the status of a robotic limb in 2-DoFs configuration: one encoding the Cartesian coordinates of the end-effector of the robotic arm (i.e., *Task-space feedback*) and another and encoding the robot joints angles (i.e., *Joint-space feedback*). Feedback was delivered to blindfolded participants through vibrotactile stimulation applied on participants’ leg. After a 1.5-hour training with both feedbacks, participants were significantly more accurate with *Task* compared to *Joint-space feedback*, as shown by lower *position* and *aiming errors*, albeit not faster (i.e., similar *onset delay*). However, *learning index* during training was significantly higher in *Joint space feedback* compared to *Task-space feedback*. These results suggest that *Task-space feedback* is probably more intuitive and more suited for activities which require short training sessions, while *Joint space feedback* showed potential for long-term improvement. We speculate that the latter, despite performing worse in the present work, might be ultimately more suited for applications requiring long training, such as the control of supernumerary robotic limbs for surgical robotics, heavy industrial manufacturing, or more generally, in the context of human movement augmentation.

## 1. Introduction

Research has shown the pivotal role of multimodal sensory feedback in human motor control. Indeed, while it is possible to plan and execute movements without a continuous stream of afferent information (e.g., ballistic movements), a feedback control is fundamental for many complex actions during our daily life [1, 2]. This mechanism can easily be extended to the use of tools, which not only requires a precise motor command but also substantially benefits from a reliable and easily understandable sensory feedback.

The study of advanced tools for rehabilitation, such as prostheses or manipulanda, was heavily influenced by the importance of multimodal sensory feedback, and a great effort has been made to retrieve and relay sensory information from the artificial limb to the user. Indeed, a closed-loop system involving the user and the artificial limb improves its motor control [3–5] and the acceptability of the artificial device into body representation (i.e., embodiment) [6–8]. The brain possesses multiple representations of the body [9], created through multisensory integration [10]. It is known that consistent and repeated interactions with a tool can alter body representations [11], hence the use of a device such as a prosthesis within a closed-loop system could possibly lead to its embodiment [12, 13]. The overlap between brain areas involved in embodiment and motor control [14–16], and studies on bidirectional prostheses in which multimodal sensory feedback improves both ownership and control at the same time [5, 7, 17, 18], support the hypothesis that embodiment of a tool could improve its skilful use.

These benefits could most likely be applied not only to restorative devices (i.e., prosthesis) but to augmenting device as well, such as robotic limbs that can help healthy users in carrying on complex (e.g., robotic surgery) or dangerous (e.g., hazardous material handling) activities by improving their abilities while sharing the same workspace. The possibility of increasing physical capabilities beyond those of a healthy human being is at the centre of the emerging field known as human movement augmentation (HMA). In the context of HMA, Supernumerary Robotic Limbs (SRL) caught the attention of scientific community. Indeed, SRLs are robotic devices (e.g., arms or fingers) that can be used together with natural limbs to enable tri-manual tasks [19, 20] and can open up new ways to physically interact with the environment, mainly thanks to the Degrees of Freedom (DoFs, i.e., allowed movements and orientations in space) they possess [19].

The SRLs currently available are prototype solutions, mainly employed in structured environments, (e.g., research laboratories), to support human body and minimize load and to guarantee user safety and stability during working activities (e.g., industrial manufacturing) [21–25], but they could eventually be employed in many other scenarios (i.e., robotic surgery, rescue operations or dangerous materials manipulation) [12].

Despite the potential benefits of multimodal sensory feedback, the process of collecting, encoding and relaying SRL feedback to users received relatively little attention. Indeed, the majority of studies which employed SRLs simply relied on incidental feedback (e.g., constant visual monitoring), without exploiting supplementary feedback (e.g., vibrotactile stimulation encoding robot status) [24, 26–30]. However, this approach is problematic for several reasons. Visual feedback is highly reliable but slowly processed compared to other sensory modalities, such as somatosensory stimuli [31]. Additionally, a constant visual monitoring of the SRL requires a great amount of attention, thus constituting a cognitive burden [32–34], while somatosensory feedback can be delivered in a less intrusive and discreet way, and belongs to the same sensory modality as the SRL information it relays, making it easier to process by our cognitive system. Finally, vision could be occluded for several reasons in a real-life scenario (e.g., environment conformation or task features). Hence, determining which alternative source of information could properly supplement visual feedback is a pressing matter. Some authors investigated the possibility to convey the status of a SRL through several forms of haptic feedback: pressure against a user’s body part [35, 36], electrotactile [37, 38] and vibrotactile stimulations [39–42]. Results obtained in these studies generally showed an improvement of user SRL control, in terms of force regulation, accuracy and completion time, when they received supplementary feedback compared to control conditions (e.g., only visual feedback or somatosensory feedback with irrelevant or non-meaningful content, depending on the specific study).

While these works agree on exploiting somatosensory modality to encode SRL supplementary feedback, they do not investigate in a systematic way the efficacy of different feedback contents (i.e., information carried by the feedback signal, such as end-effector contact force or SRL joint angle) to determine which one could lead to better control performance or could be more easily understood, in the specific framework of HMA. Indeed, the choice of the feedback content is frequently guided by knowledge related to other fields (e.g., prosthetics or teleoperation) or by task features and constraints. However, results obtained in other fields might not always be well suited for HMA for several reasons: i) while intuitive and easy-to-process feedback is always desirable, in the case of HMA it is even more critical, since SRL supplementary feedback and natural bodily feedback will have to be processed simultaneously; ii) supplementary feedback should not interfere with the activity of other limbs, so the location for supplying feedback is particularly important; iii) it cannot exploit substitutive neural substrates, as it happens in the field of prosthetics (e.g., peripheral severed nerves), but it requires new neural resources [43]. Hence the need to study supplementary feedback with specific reference to HMA.

To achieve this objective, we previously [39] investigated the impact of SRL end-effector Cartesian position feedback compared to SRL joint torques, by asking blindfolded participants to replicate offline (i.e., after the feedback signal ceased) the position of the SRL end-effector while relaying only on supplemental feedback. Results suggested that Cartesian space position can be more easily exploited by users, which obtained lower completion time and higher accuracy, compared to joint torques feedback. However, the SRL target applications require the ability to exploit supplementary feedback online.

Thus, in the present work we employed an experimental platform which possesses key HMA features (i.e., robot and user share the same workspace [40], feedback is delivered on a limb not involved in the task), to compare two different feedback contents related to the position of the robotic arm in an online 2D tracking task. Both feedback signals were coded using vibrotactile stimulation, which is easily implementable and able to relay meaningful information [44–46]. However, different features were encoded to relay the position of the robot: either the Cartesian coordinates of the robot end-effector in the 2D space (*Task-space feedback*) or its joints angles (*Joint-space feedback*).

Understanding which of these two different strategies performs better gives clues also on another main question: do users consider the robotic arm as an external object or as a part of their body? Indeed, on one side several studies have proven that the brain uses extrinsic reference frames to evaluate the position of objects in the external world [47, 48] and execute goal-oriented movement, suggesting that Task-space feedback might be more performing if the robotic arm is treated as an external object. Conversely, other works show that to build the body schema [49, 50], the brain mainly relies on intrinsic proprioceptive-related reference frames, such as joint features [51, 52]. This suggests that if the robotic arm gets included in the body schema, and thus treated as belonging to the body, Joint-space feedback could outperform Task-space feedback when detecting EE position. Moreover, joint angles are often exploited in robotics to describe the whole kinematic chain of the robot for learning its body schema when multiple DoFs are enabled [53, 54]. In the present work, blindfolded participants had to replicate the 2D position of the end-effector online by relying exclusively on either *Task* or *Joint-space feedback*, tested in two different sessions, after a 1 hour training. We reckon that closing the loop between human and supernumerary robotic arm is a complex matter and despite its importance in the HMA framework, we decided to address first the issue of sensory feedback. Hence, in the present work the robotic end-effector moved autonomously. This allowed us to obtain more generalizable results that are not strictly related to a specific control strategy, and to avoid the detrimental or confounding impact on sensory processing due to any imperfection in the supernumerary robot control.

## 2. Materials and methods

### 2.1. Experimental setup

**Fig 1:**
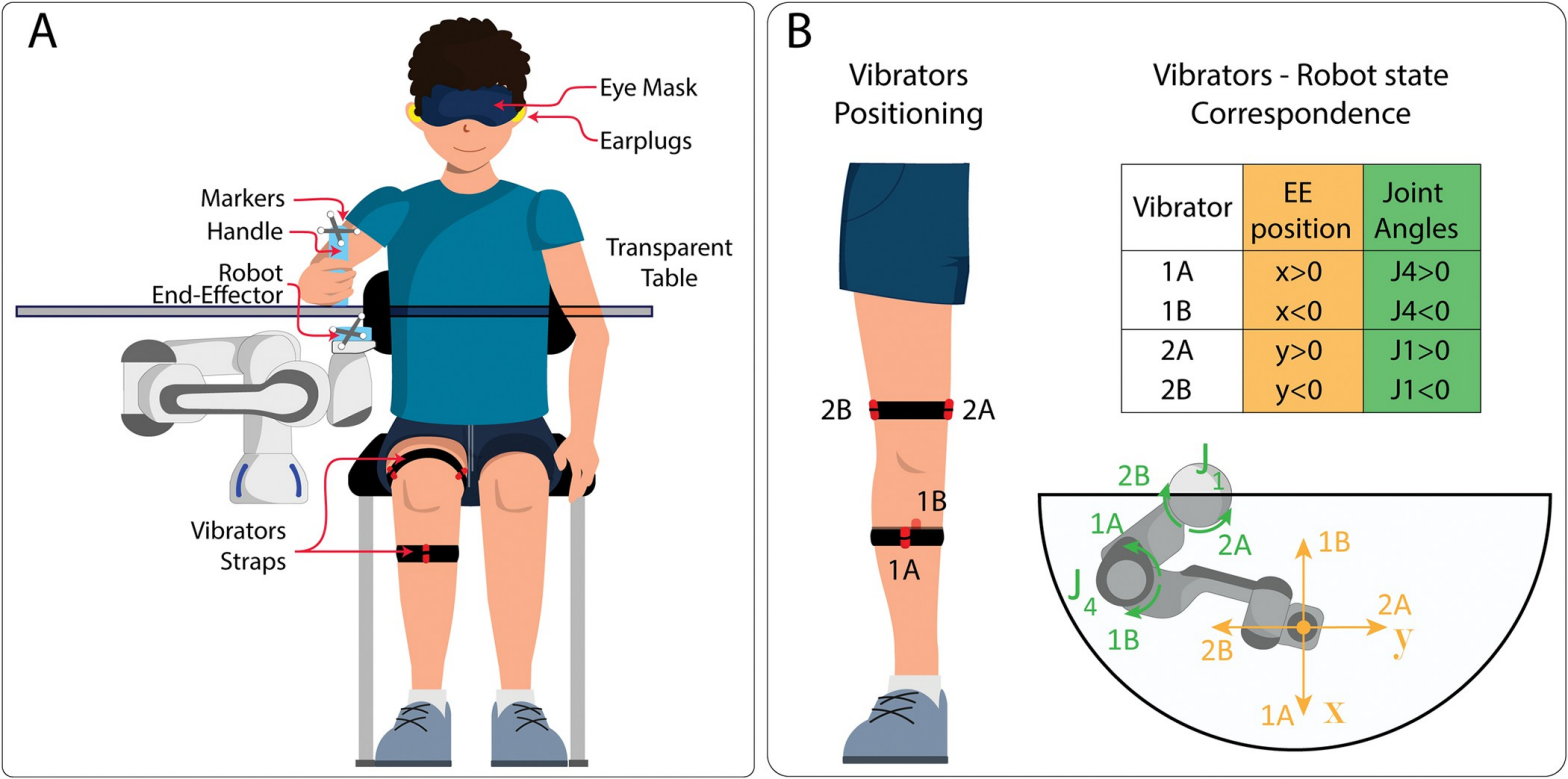
Experimental setup and vibrator positioning. **Panel A:** Participants sat medially to the robot and held a custom plastic handle with their right hand. Another custom plastic support was attached to the robot EE. Passive markers were attached to both handle and support to track them using infrared cameras. Participants moved the handle upon the transparent table while the robot EE moved below the table. The handle was equipped with a soft pad to reduce friction with table surface. Participants wore disposable earplugs to mask the noise of the robot moving, and could be blindfolded to remove visual feedback, depending on the experimental block. **Panel B**: A total of four vibrators were worn by participants: two of them on the medial (2A) and lateral aspect (2B) of the thigh, about 12 cm proximal to the knee; the other two on the frontal (1A) and posterior aspect (1B) of the calf about 20 cm proximal to the ankle. Vibrators were placed to be in touch with soft tissue and avoid bone crest. The matrix showing information conveyed by vibrators in each condition is shown on the right. X and y represent the EE coordinates with respect to the workspace centre, while J1 and J4 represent the first and fourth robot joint angles, as represented in the lower part of panel B. Positive and negative values of x, y, J1 and J4 are considered with respect to the starting configuration of the robot, with the EE located in the centre of the workspace (x, y, J1 and J4 equal to 0). The lower part of panel B shows which vibrators were activated when joints rotated (Joint-space feedback, green) or when the EE moved away from the workspace centre (Task-space feedback, yellow).

Participants sat in front of a transparent table, wore earplugs for the duration of the experiment and kept their right shoulder aligned with the centre of the workspace, as well as the end-effector of a robotic manipulator (Panda robot by Franka Emika GmbH) that was positioned below the participant’s arm (Fig 1A). This positioning was chosen to have the robotic arm located in parallel with the natural upper limb but operating on a lower plane (2 cm below the surface of the table) and maintaining the same eye-robot relation of a natural limb (first person perspective).

Only two robot’s DoFs (first and fourth joints) were employed to make planar movements, chosen to first test the feedback in a simple 2D task. The robot tip, namely the end-effector (EE), was clearly visible through the transparent table. The robot was controlled with a custom interface developed in C++ language, using Qt libraries and running on Ubuntu 16.04 O.S.

**Fig 2:**
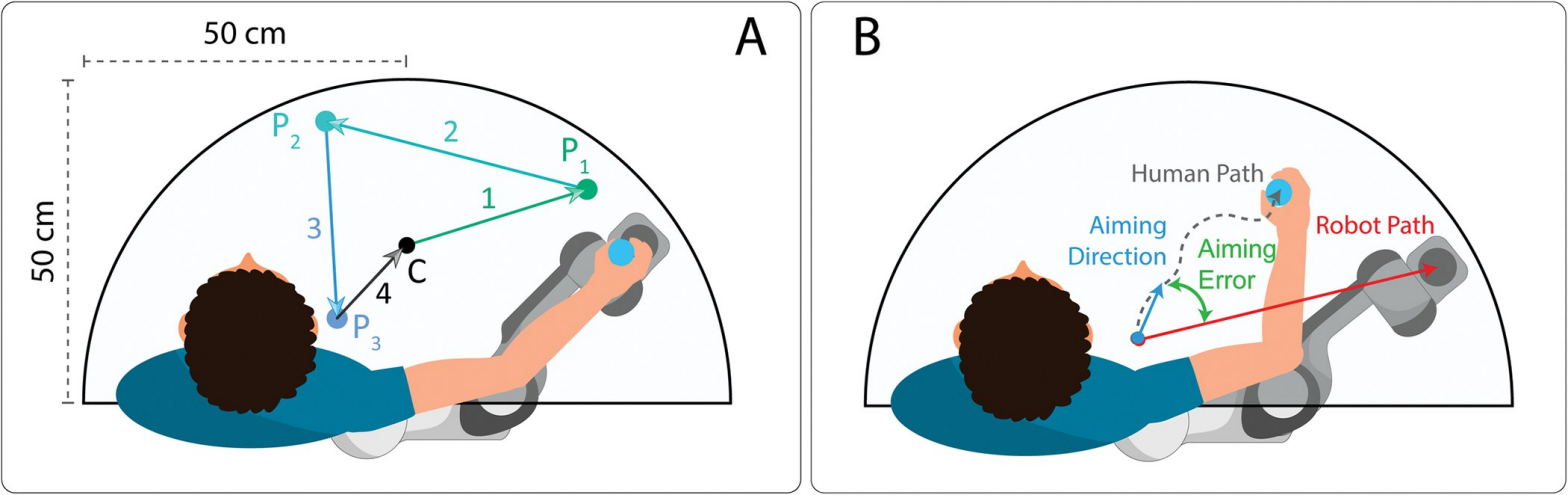
Representation of the half-circle shaped workspace. **Panel A.** P1, P2 and P3 represent three sequential, randomly generated, target points. C represents the centre point, which is both the starting and the ending position of a closed path. Lines 1, 2, 3 and 4 represent straight motions of the robot from one point to another. **Panel B.** Aiming direction (light blue) of the participant computed as the vector linking the starting point and the average 25% of the whole human path (dotted grey), robot path (red) and aiming error (green) computed as the angle between human and robot direction vectors.

Participants moved their right arm onto the table’s plane while holding a custom printed plastic handle that could slide on the table with minimal friction. Both robot and participants moved within a half-circle shaped workspace with a radius of 50 cm (Fig 2). Passive reflective markers were attached to both robot EE and plastic handle to track their movement through two infrared cameras (PrimeX13W by Optitrack) placed in front of the setup. Motion tracking data were recorded and saved at the frequency of 240 Hz.

Somatosensory feedback was relayed using four vibrator motors (Model: 307–103 by Precision Microdrives Inc.) attached to cloth strings through custom made supports, and placed on participants’ right leg, which was not involved in the task, to avoid any interference with natural limb proprioception, during arm movements. More specifically, two vibrators were placed on the medial (2A) and lateral aspect (2B) of the thigh, and the other two on the frontal (1A) and posterior aspect (1B) of the calf (see Fig 1B). Before starting the experiment, vibrators’ placement was slightly adjusted for each participant and fine-tuned to clearly perceive the stimulation of each motor. Vibrators were controlled through a custom Printed Circuit Board [39]. The stimulation amplitude and frequency were coupled with parameters extracted from the robot kinematic data read in real time (see section Somatosensory Feedback for a detailed description) and modulated by the microcontroller (STM32F446 by STMicroelectronics Inc.) embedded on the PCB.

### 2.2. Somatosensory feedback

The state of the robot was translated into input to control vibrating motors using two different feedback strategies: *Task-space* or *Joint-space* position. In both cases, a linear mapping between the rated voltage of the vibration motors and the robot state has been adopted:

$$V_{ij}\;=\;V_{ij}^{min}\;+\left|\;\psi_{i}\cdot (\frac{V_{ij}^{max}\;-V_{ij}^{min}}{\;\psi_{i}^{max}})\right|$$ (1)

Denoting with *V*_*ij*_ the rated voltage of the *i*⋅*j*-th (*i* = {1,2} and *j* = {*A*, *B*}) motor, *ψ*_*i*_ the *i*-th element of the feedback type considered and $\psi_{i}^{max}$ its maximal admissible value. In all cases, $V_{ij}^{min}$ was set to 0.2 V (minimal value to guarantee the vibration motors to be activated as soon as *ψ*_*i*_ becomes different from zero) and $V_{ij}^{max}$ was set to 3.6 V (maximal supply voltage). It is important to note that in this type of vibrator motors amplitude and frequency are linked together, and by increasing the power supply tension they both increase, hence we will, from now on, refer to vibration intensity.

In *Task-space feedback* condition, participants received information concerning the position of the robot EE in a planar space, expressed in cartesian coordinates *(ψ*_*1*_ = *X*_*EE*_ and *ψ*_2_ = *Y*_*EE*_). Motors 1A-1B and 2A-2B coded the *X*_*EE*_ value (proximo-distal position) and the *Y*_*EE*_ value (latero-medial position) respectively. *X*_*EE*_ and *Y*_*EE*_ were equal to 0 in the workspace centre. Participants received a continuous vibratory stimulation as long as the robot EE was not in the workspace centre, and the vibration intensity was linearly modulated according to the distance between EE and centre.

Conversely, in *Joint-space feedback* condition, motors 1A-1B (*ψ*_1_) and 2A-2B (*ψ*_2_) linearly encoded for the amplitude of the J_4_ (elbow-like) and J_1_ (shoulder like) joint angle, respectively. Both angular positions were equal to 0 for joints configuration corresponding to the EE in the workspace centre.

In both feedback conditions, vibrators A and B delivered positive and negative encoded values respectively (Fig 1B), and the vibration ceased between trials, to avoid sensory adaptation. It is important to note that the feedback approach used in this study is focused on robot position (in Cartesian or angular terms) rather than robot motion. Hence, despite providing different content, the two feedback signals (i.e, *Task-space* and *Joint-space*) ultimately provide position feedback. Indeed, the planar configuration of the robot with all joints locked excluding J1 and J4 (Fig 1A and 1B), prevents redundancy and assures that EE position in the Cartesian space is univocally defined in the joint space and vice versa.

### 2.3. Experimental protocol

Twenty participants (aged 25±4 years, ten females, all right-handed as assessed through Oldfield test) [55] took part in the study, but three of them were discarded because of missing data due to technical issues. They were enrolled after signing a written informed consent, and experimental procedures were approved by the Ethics Committee of the Università Campus Bio-Medico di Roma (EMBODY protocol) and carried out according to the Declaration of Helsinki and future amendments.

Participants underwent two experimental sessions, each one divided into six blocks. During each block, the robot moved autonomously from one point to another, within the workspace, and participants were asked to follow the robot EE as closely, accurately and quickly as possible (i.e., without waiting for the robot to complete its movement). Participants had to slide the handle upon the table to match the robot EE position, without rising if from the table surface.

Robot movements were organized in 97 closed paths, starting from and ending in the workspace centre. Each closed path was made of four sequential motions (straight lines), connected by four different target points, last one being the workspace centre (Fig 2A). To generate realistic and heterogenous trajectories, each robot motion had a duration that could range randomly from 1.5 to 3.5 seconds and a random travel distance, constrained within 10 cm and the entire human-robot shared workspace. Each experimental trial started with the onset of the robot motion and ended when participants stopped moving the handle, and after that a new trial begun.

**Fig 3:**
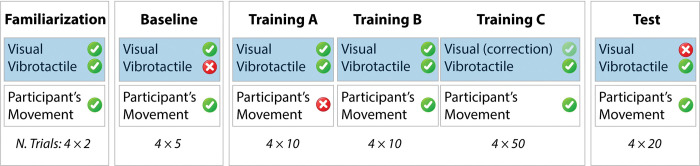
Experimental protocol. “Visual” and “Vibrotactile” refer to the type of feedback relayed to participants (blue shade), while “Participant’s Movement” refers to participants’ arm movement which followed robot motions. Numbers below experimental blocks indicate the number of trials and express how many times the closed paths of 4 sequential motions were repeated within a specific block. Green ticks denote presence of a feedback type/movement, while red marks denote their absence. Green semi-transparent tick in Training C denotes presence of Visual feedback only at the end of each motion, to allow the correction of the position.

The two experimental conditions, namely *Task-space* and *Joint-space feedback*, were tested during two different days, roughly one week apart, in a counterbalanced order, so that half of participants performed *Task-space* condition first while the other half started with Joint-space condition, to control for any possible order and carryover effect. Each experimental session was made by the following six different phases (graphically represented as blocks in Fig 3):

- *Familiarization (2 closed paths*, *8 motions*, *5 minutes)*: participants were required to follow the robot EE while receiving both visual and vibrotactile feedback, to become familiar with the experimental setup.
- *Baseline (5 closed paths*, *20 motions*, *5 minutes)*: participants were asked to follow the robot EE with full visual feedback, without receiving vibrotactile feedback that, in the absence of proper training, could act as a disturbing factor. This block was used to collect participants’ baseline performance in each condition.
- *Training A (10 closed paths*, *40 motions*, *10 minutes)*: at this stage, participants received visual and vibrotactile feedback, without making any movement, in order to focus exclusively on the association between vibration and robot motion. This block was used to let participants understand the meaning of the two feedback types, allowing them to couple the vibrotactile stimulation with robot activity, employing action observation strategies.
- *Training B (10 closed paths*, *40 motions*, *10 minutes)*: participants were asked to follow the robot EE, while receiving visual and vibrotactile feedback. This block aimed at reinforcing with action execution the association between vibration and robot movement established during Training A, as it was demonstrated that active movements facilitate associative learning [56].
- *Training C (50 closed paths*, *200 motions*, *50 minutes)*: participants were asked to follow robot EE motions, while keeping their eyes closed, relying only on vibrotactile feedback, and to open their eyes only when the vibratory feedback ceased, at the end of the robot motion. This block had the two-fold objective of introducing the absence of visual feedback (this justifies its high number of repetitions) and, eventually, giving a direct visual confirmation to participants regarding their blindfolded performance, allowing corrections and thus completing the association process. Additionally, the correction of the final position prevented propagation of drift error resulting from inaccuracies in the replication of sequential motions.
- *Test (20 closed paths*, *80 motions*, *20 minutes)*: participants followed the robot EE without visual feedback (blindfolded) for the entire duration of the task and relied exclusively con vibrotactile feedback. To avoid error propagation in the absence of vision, the experimenter manually re-positioned the subject’s handle in the workspace centre after the fourth motion of each closed path (i.e., after participants moved from P_3_ to C, see Fig 2A).

The three training phases A, B and C were employed to progressively reduce visual feedback and likely increase the reliance of participants on vibrotactile feedback concerning the robot state. Overall, each experimental session lasted roughly 2 hours, preparation included.

### 2.4. Data analysis

Data were processed using Matlab 2020a and statistical analysis was performed using JASP 0.16.0.0. Participants’ performance was assessed for both feedback conditions (*Task* and *Joint-space feedback*) before training (*Baseline block)*, during *Training C* and after training *(Test block)*. We evaluated five indexes in different blocks:

- *Position error* (evaluated during *Baseline* and *Test*): computed as the Cartesian distance between handle and robot EE on the plane, calculated for each time sample and then averaged along trials.
- *Onset delay* (evaluated during *Baseline* and *Test*): computed as the time between the start of the robot motion and the start of the participant motion for each trial.
- *Delay-compensated position error* (evaluated only during *Test*): computed by using *Onset delay* to shift in time the position of the participants’ handle. This was done to account for the delay in the participant’s movement onset and obtain a more accurate estimation of the error between the robot EE and participants.
- *Aiming error* (evaluated during *Baseline* and *Test*): defined as the angle between the aiming movement direction of the participant and the robot. We considered the participant aiming direction as the intended direction of movement, thus the one corresponding to the initial part of hand movements prior to deviations or final adjustments. To do this, we considered the whole curve described by the hand during one trial and the aiming movement direction was computed as the vector connecting the starting point to the point corresponding to the 25% of the participants’ whole movement (to mitigate the effect of jerky movements, the 25% point was calculated as the average point in the interval between the 20% and the 30% of the whole movement) (Fig 2B). Since the robot moved on linear paths, its movement direction was computed as the vector connecting the starting and ending points in each straight movement. Aiming Error (AE) was evaluated as follows:

$$AE=\cos^{-1}\frac{{\vec{d}}_{r}\cdot {\vec{d}}_{h}}{\left\|{\vec{d}}_{r}\|\|{\vec{d}}_{h}\right\|}\;[deg],$$ (2)

where ${\vec{d}}_{r}$ and ${\vec{d}}_{h}$ denote the movement direction of robot EE and participant’s handle respectively.

v. *Learning* (evaluated only during *Training C*): defined as the slope of the linear regression evaluated on the average position error of each closed path over time. Negative values correspond to performance improvement (i.e., error reduction over time).

Data recorded in *Test* and *Training C* were corrected (normalized) according to the baseline performance by subtracting, for each participant, their *Baseline* average *position error* and *onset delay* from their corresponding values in the *Test* block.

*Joint* vs *Task-space* comparison was run for every index, using Student’ t test with normally distributed data and Wilcoxon signed-rank test with non-normally distributed data. No statistical corrections were applied since we did not perform multiple comparisons.

## 3. Results

Among the 17 participants, two were excluded from the analysis because the absolute average value obtained in at least two indexes (e.g., position error, aiming error, onset delay) by those participants exceeded the grand average by more than 2 standard deviations. See Supplementary Materials (S1 Fig) for plots including all 17 participants.

*Position error*, *onset delay*, *delay-compensated position error* and *learning* data were normally distributed (Shapiro-Wilk all p>0.111, all W>0.904) while *aiming error* data were not normally distributed (Shapiro-Wilk p<0.001, W = 0.717).

**Fig 4:**
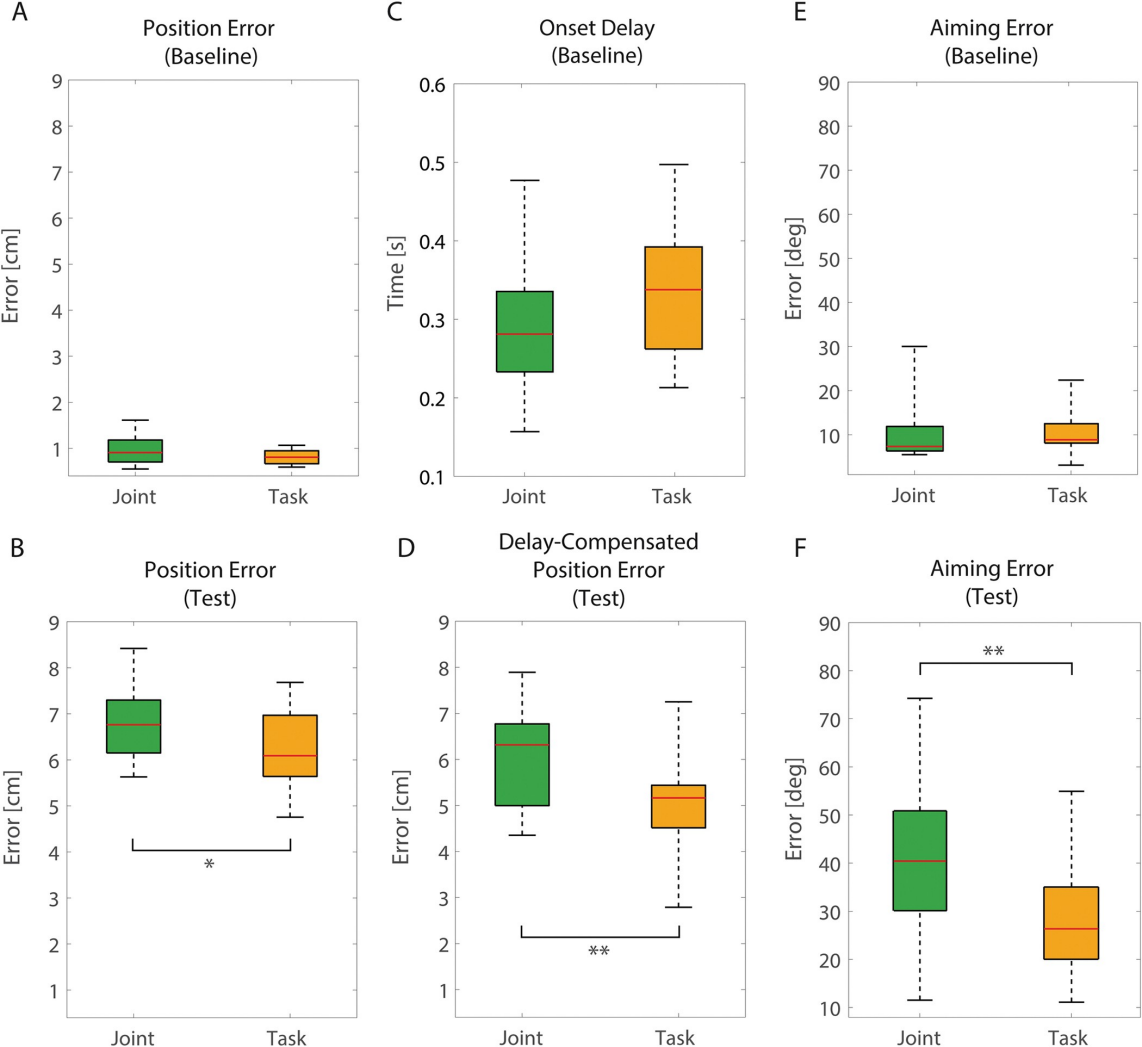
Position error, onset delay and aiming error. Position error obtained by participants during baseline (A) and test block (B), onset delays obtained in baseline (C), delay compensated position error obtained during test (D) and aiming error obtained during baseline (E) and test block (F). Joint-space feedback condition is shown in green while Task-space feedback condition is shown in orange. Red line represents median values. * denotes p<0.05, ** denotes p < 0.01.

Concerning *position error*, no significant difference emerged between *Joint vs Task-space feedback* condition (0.9±0.3 cm vs 0.8±0.1 cm; p = 0.086, t = -1.847, df = 14) in *Baseline* block (in which no vibratory feedback was relayed). Conversely, in *Test* block, participants obtained significantly higher position error during *Joint vs Task-space feedback* (6.8±0.8 cm vs 6.1±0.9 cm; p = 0.027, t = 2.460, df = 14) and this effect is even more pronounced considering delay compensated position error (6.0±1.1 cm vs 5.0±1.2 cm; p = 0.006, t = 3.254, df = 14) (Fig 4A, 4B and 4D).

Accordingly, *aiming error* shows no significant difference between *Joint vs Task-space feedback* in *Baseline* (11.4±7.9 deg vs 10.2±4.7 deg; p = 0.679, W = 52, z = -0.454), whereas in *Test*, *aiming error* was significantly higher in *Joint vs Task-space feedback* (41.2±16.1 deg vs 28.4±11.9 deg; p<0.001, W = 115, z = 3.124) (Fig 4E and 4F).

Concerning *onset delay*, no significant difference emerged between *Joint vs Task-space feedback* neither in *Baseline* block (0.29±0.09 s vs 0.33±0.07 s; p = 0.268, t = -1.152, df = 14) nor in *Test* block (1.68±0.71 s vs 1.40±0.66 s; p = 0.202, t = 1.339, df = 14) (Fig 4C).

**Fig 5:**
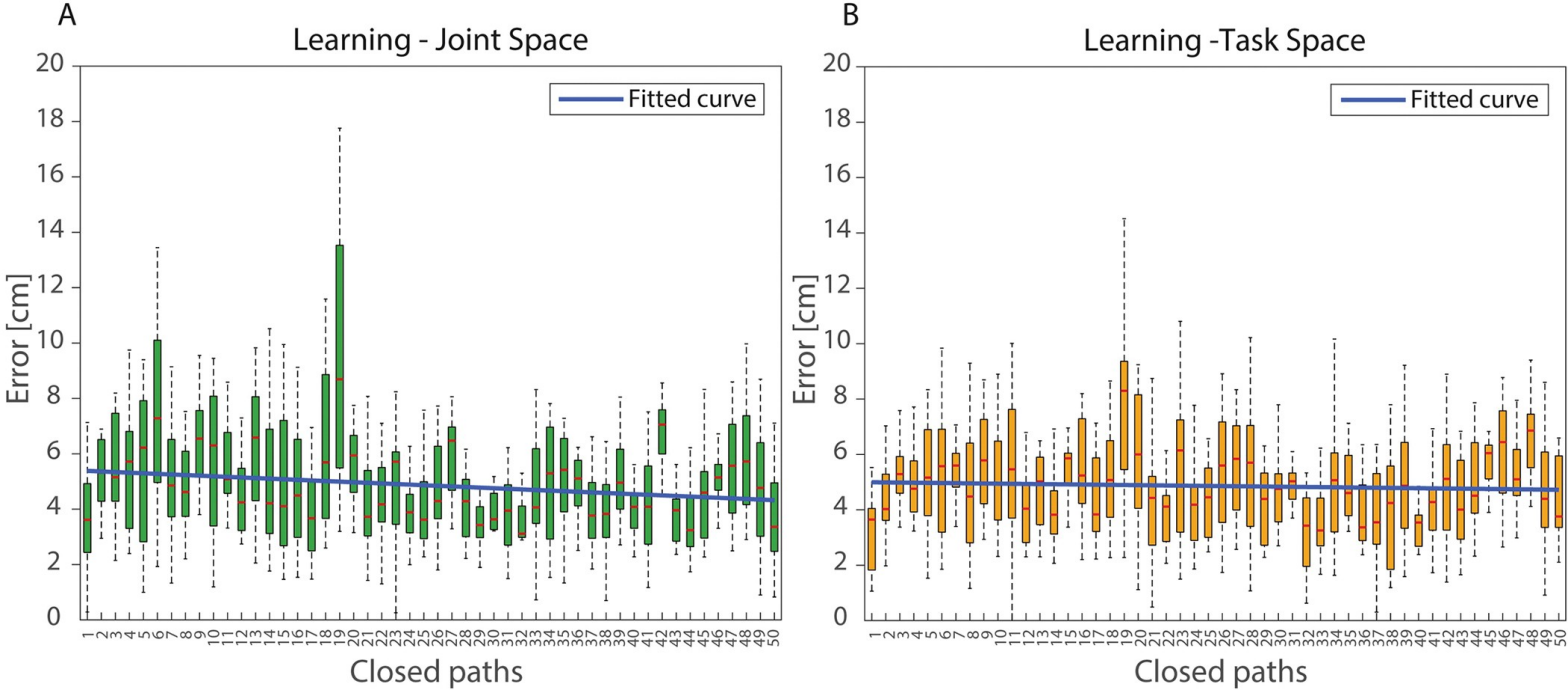
Learning index. Each bar represents the position error obtained by all participants during a single Closed path, computed as the mean of the position error of four sequential motions. Red lines represent median value and bars are filled within 25^th^ and 75^th^ interquartile range. Panel A shows learning index during Joint Space (green) condition while Panel B refers to Task Space (orange) condition. Blue lines represent the linear regression.

Finally, during *Training C* block, the slope of the linear regression, representing *learning index*, was significantly steeper in *Joint vs Task-space feedback* (-0.0034 cm/closed path vs -0.0005 cm/closed path; p = 0.023, t = -2.539, df = 14) (Fig 5).

## 4. Discussion

The present work investigates for the first time the efficacy of two different feedbacks, namely *Task-space* and *Joint-space feedback*, in conveying the 2D position of a robotic arm, sharing the workspace with the user upper limbs, during an online tracking task.

Participants followed the robot EE in the absence of visual feedback with higher accuracy (significantly lower *position error*, *delay-compensated position error* and *aiming error*) when they relied on *Task* compared to *Joint-space feedback*. However, participants required similar time to initiate chasing movements in both conditions (no significant difference in *onset delay*). Since *onset delay* can be considered as an implicit measure related to cognitive load [57, 58], these results suggest that the computation of both feedbacks requires similar amount of neural resources in a given time. Overall, in our experiment, *Task-space feedback* proved to be more effective, as it allowed to reach better performance with the same cognitive effort. However, during *Joint-space feedback* training, accuracy showed a higher improvement over time compared to *Task-space feedback* training.

Hence, *Task-space feedback* is certainly a better choice for tasks in which a lengthy and potentially costly training is not feasible.

It might be possible that our current approach created a bias toward Cartesian space, due to the simpler information coding (i.e., vibration delivered along two perpendicular axis). However, we deem unlikely that this could have influenced results since this possible bias would have been balanced by the proximo-distal correspondence in the coupling between robot frame and vibrator positions in Joint-space condition. Alternatively, a simpler coding for the Joint-space feedback could have been a rotational stimulation pattern (i.e., a dynamic stimulation which moves on the participants skin according to the Joint rotation). However, this would have produced a motion-based feedback (i.e., informative only as long as the robot was moving) instead of a status feedback (i.e., informative both in dynamic and static condition). Additionally, applying the same approach to Task-space feedback would have required a workspace-related interface (e.g., mapping the entire workspace through a matrix of vibrators applied on participants’ skin or having a series of vibrators activate sequentially to mimic the motion of the EE).

Further comments on our results can be made if we draw a parallel between our experimental protocol and the real-life scenarios in which robotic limbs are likely to be employed, especially when framed in the HMA scenario. Indeed, handling of dangerous materials or precise robotic surgery are target applications which imply i) the presence of visual feedback and ii) a long and task specific training before committing to the real-life activity.

Concerning the first point, in the present work we chose to perform our tests in the absence of vision to allow even subtle differences to produce changes of performance, which otherwise could have been hidden by the massive contribute of vision. As can be seen in Fig 4, *Task-space feedback*, despite being the one performing better, still shows a considerable position error (~5 cm). We deem this acceptable, since the supplementary vibrotactile feedback is eventually not meant to completely substitute vision, but to complement it. Indeed, the estimation of the robotic limb status through vibrotactile feedback may reduce the reliance on visual feedback, grant additional information that cannot be appreciated through vision because of their nature (e.g., contact force) or environmental features (e.g., momentarily obstructed vision) and allow users to better perform motor tasks.

Concerning the length of training, we set it to test each condition in a single day while avoiding fatigue in participants. However, in light of the present results, we argue that next studies should implement a training of several days or weeks, so that the best performing feedback would also be well suited for SRL target activities requiring highly professional and trained users.

Some considerations suggest that *Task-space feedback* is more intuitive and that a longer training could have changed our results related to which feedback performs better: i) we are accustomed to estimate the position of external objects (robot EE, in our case) through vision, which maps surrounding space using Cartesian-space coordinates [59], and ii) locate the position of an object in space through angles, as required in the Joint feedback condition, might be particularly difficult at first since angle amplitude and distance could be not linearly coupled. Indeed, *learning index* suggests that *Joint-space feedback* might outperform *Task-space feedback* in the long run.

*Onset delay* shows that, despite being instructed to follow the robot EE as closely as possible in space and time (i.e., online), participants always presented a certain delay (~1,5 s) before initiating the tracking movement during *Test block*. This time window can be divided in three parts. The first part of the delay is due to the time required for the computer to generate the feedback signal (i.e., information collected from the robot, encoded into a vibratory pattern and delivered to participants), and for the participant to sense it (i.e., receptors activated by the stimulation and somatosensory information conveyed to primary sensory cortex). This delay can be roughly quantified as lower than 100 ms. About 300 ms is the time needed to initiate simple reaction movements (i.e., simple reaction time task) [60, 61], and accordingly this delay was also present in the *Baseline*, when chasing movement were guided by full visual feedback (Fig 4). The remaining ~1.1 s is likely the time needed for the computation of the supplementary feedback. Indeed, participants were required to infer EE Cartesian position or robot’s joint angle from a vibratory pattern; in other words, they had to gather proprioceptive-related information from a tactile stimulation. This translation across modalities required by our approach (i.e., cross-modal stimulation paradigm) [44] most likely demanded additional computational steps from the. To decrease the time required to decode supplementary sensory feedback, an interesting strategy would be to remove the need for a decoding process: instead of using an heteromodal stimulation, proprioceptive information could be delivered by acting directly on muscle spindles [45, 62] or skin stretch mechanoreceptors [63], thus generating an illusionary sensation of movement.

Summing up, with the present study, we demonstrated that when the training of the robotic limb supplemental feedback decoding is restricted to roughly 1.5-hour, *Task-space feedback* is more effective in terms of accuracy, compared to *Joint-space feedback*, probably because the former is more intuitive and can reach its full potential after a relatively short training. While we previously demonstrated that *Task-space feedback* is more informative than a random stimulation pattern, used as control [39], position error remained consistent in the present work. Thus, future studies should investigate whether such feedback would be useful in a real-life scenario where vision is available; alternatively, visual feedback could be gradually impaired to determine how unreliable vision must be [64] in order to enhance performance through supplementary feedback.

Moreover, despite being the less performing option with short training, *Joint-space feedback* still shows potential for long-run performance, and we reckon it is worth further investigation. Indeed, we speculate that coding robotic limb feedback using a language inspired by human physiology in the representation of the body schema [49, 50], could eventually be more suited particularly for the HMA framework, in which the user is supposed to control and feel the SRL as a real limb. Concerning this last point, future studies should implement the present findings in a closed-loop system to explore the mutual impact that motor control of a robotic limb and its supplementary sensory feedback might have on each other.

Finally, results obtained in this work could also benefit fields such as prosthetics, where feedback on the position of the robotic arm is also a key feature to improve control performance.

## Supporting information

S1 Fig Position error, onset delay and aiming error including outliers. Position error obtained by 17 participants during baseline (A) and test block (B), onset delays obtained in baseline (C), delay compensated position error obtained during test (D) and aiming error obtained during baseline (E) and test block (F). Joint-space feedback condition is shown in green while Task-space feedback condition is shown in orange. Red line represents median values. Black vertical lines represent 2 Standard Deviations. Dots which fall outside of 2 Standard Deviations are considered outliers participants. These plots are reported here for visual inspection only, and do not represent the analysed dataset.
(TIF)
