# Supplementary figures and images for: Comparing end-effector position and joint angle feedback for online robotic limb tracking
Source: PLoS One. 2023 Jun 8;18(6):e0286566. doi: 10.1371/journal.pone.0286566 (PMC10249844; doi:10.1371/journal.pone.0286566)

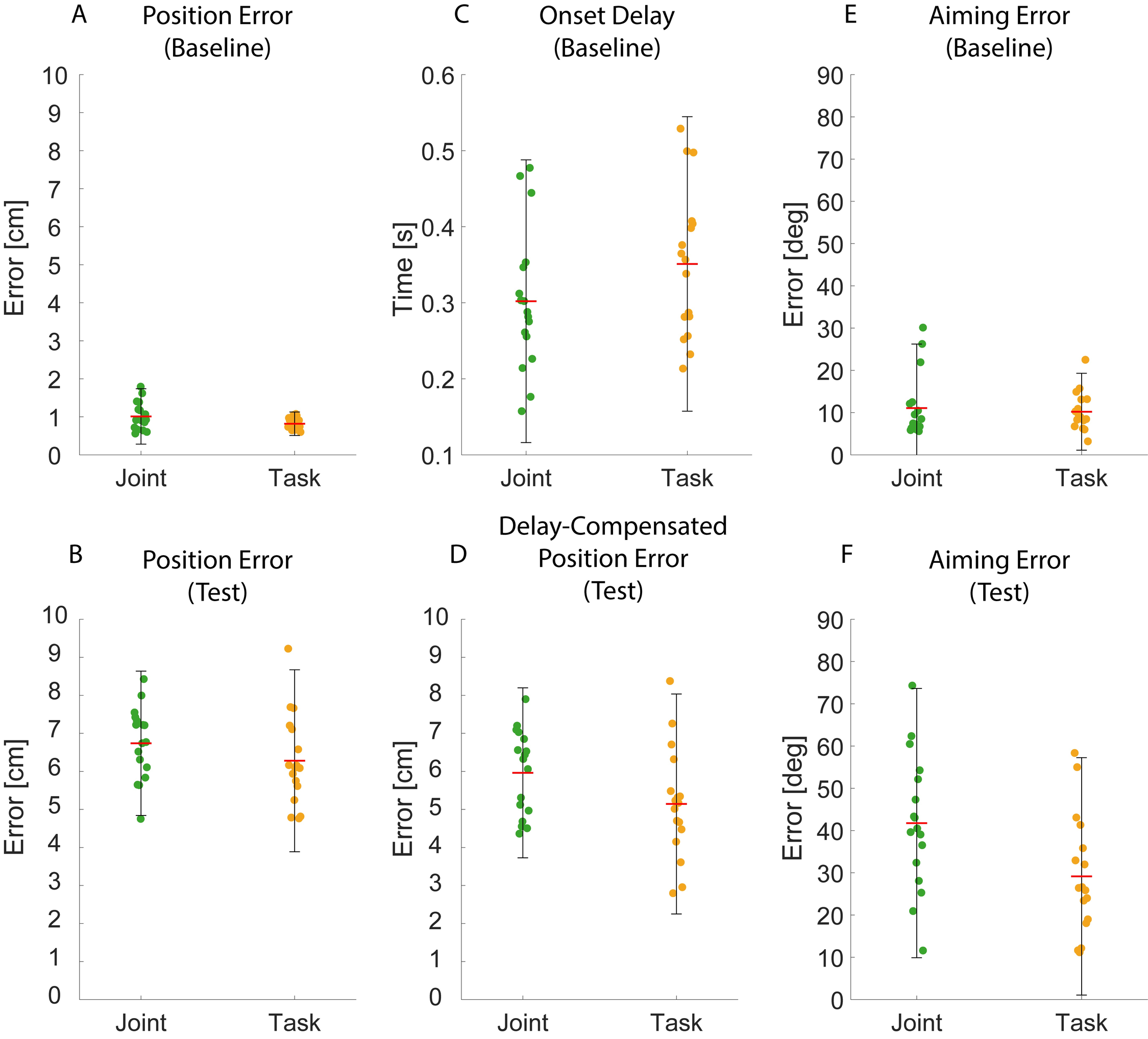

Supplement: S1 Fig — Position error obtained by 17 participants during baseline (A) and test block (B), onset delays obtained in baseline (C), delay compensated position error obtained during test (D) and aiming error obtained during baseline (E) and test block (F). Joint-space feedback condition is shown in green while Task-space feedback condition is shown in orange. Red line represents median values. Black vertical lines represent 2 Standard Deviations. Dots which fall outside of 2 Standard Deviations are considered outliers participants. These plots are reported here for visual inspection only, and do not represent the analysed dataset. (TIF) [file pone.0286566.s001.tif]
